# An examination of the association between infant non-nutritive suck and developmental outcomes at 12 months

**DOI:** 10.1371/journal.pone.0298016

**Published:** 2024-02-05

**Authors:** Alaina Martens, Hannah Phillips, Morgan Hines, Emily Zimmerman

**Affiliations:** Department of Communication Sciences and Disorders, Northeastern University, Boston, MA, United States of America; University of Rome La Sapienza: Universita degli Studi di Roma La Sapienza, ITALY

## Abstract

**Objective:**

To determine the association between infant non-nutritive suck (NNS) dynamics at 3 months and developmental outcomes at 12 months of age in full-term infants. We hypothesized that infants with more mature NNS at 3 months, as evidence by shorter burst duration, fewer cycles per burst, cycles per minute, higher amplitude, and more bursts, would have higher (better) scores on the developmental outcomes at 12 months.

**Methods:**

This was a prospective study that utilized objective and self-report measures. A five-minute NNS sample was collected from 67 infants (54% male) at 3 months of age (average age 2.99 (0.27) months). At 12 months (average age 11.91 (0.26) months), the Development Profile-3 was administered through caregiver interview.

**Results:**

Infant NNS burst duration, cycles per burst, and cycles per minute were significantly negatively associated with the Development Profile-3 cognitive domain and general scores at 12 months. This is consistent with our hypothesis that infants who have more efficient NNS (fewer bursts and cycles) at 3 months would have higher (better) scores on the Development Profile-3 at 12 months.

**Conclusions:**

Findings from this work complement emerging research linking infant NNS with subsequent neurodevelopmental outcomes. This is the first time that these associations have been examined using a quantitative and physiologic-based measure of NNS. These results seem to indicate that specific NNS metrics, which demonstrate maturation of this complex skill, may be useful predictors of neurodevelopment later in life.

## Introduction

The neural plasticity that is afforded during early brain development provides an opportunity for interventions that could help children with neurodevelopmental disorders achieve their highest potential, provided that these developmental delays are known. Neurodevelopmental disorders may be influenced by a combination of genetic and environmental factors early in life, and can manifest in a variety of characteristics [1]. The heterogeneity of clinical phenotypes makes early detection challenging. Additionally, some forms of testing are better suited to detect specific disorders than others, and their predictive power increases as the child ages [2]. For instance, eye tracking has been found to be predictive of neurodevelopmental outcomes in children with Autism Spectrum Disorder, however few studies have established this link in children under the age of two [3]. Lack of access to early testing adds an additional barrier to early intervention. Magnetic resonance imaging (MRI) is a well-established method of assessing developmental outcomes; however, neonatal MRI is not available to a majority of families [4] and can be challenging data to attain from a young child. There is a need for more accessible, non-invasive, and objective measures that provide insight into infants’ neurodevelopmental status as early in life as possible. One such measure is non-nutritive suck.

**Fig 1:**
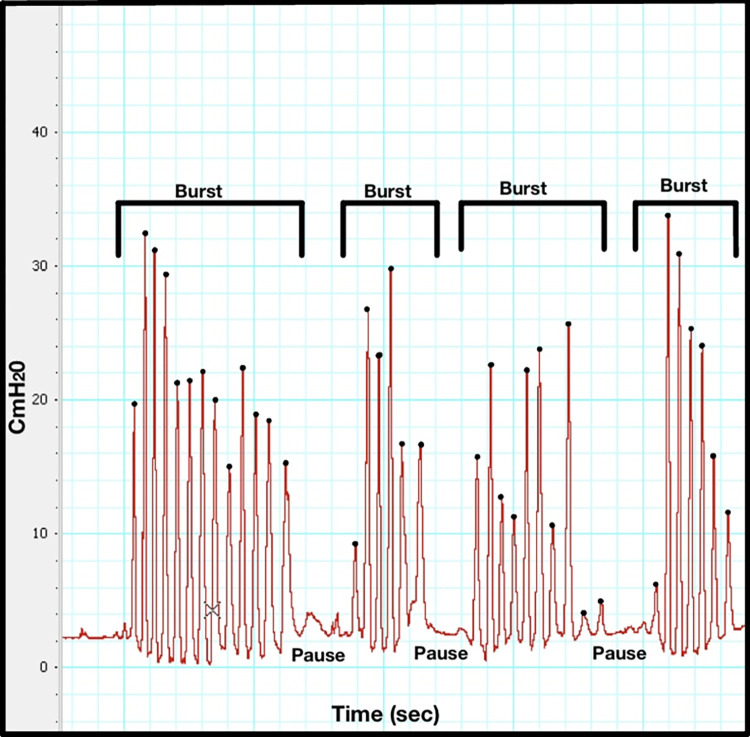
NNS Waveform from a subject utilizing our LabChart software. This infant has four bursts with 13 cycles (black dots) in burst 1, 6 cycles in burst 2, 10 cycles in burst 3, and 7 cycles in burst 4.

Non-nutritive suck (NNS), sucking without nutrient delivery, is one of the earliest motor acts an infant displays after birth, and even before birth: NNS begins to develop *in utero* at around 15 weeks’ gestational age (GA). A stable burst-pause NNS pattern is established by 34 weeks’ GA [5] with an average intra-burst frequency of 2 Hz and 6–12 suck cycles per burst (Fig 1) [6].

NNS is a crucial aspect of feeding development and can be indicative of neurodevelopmental outcomes: as many as 35–48% of infants with neonatal brain injuries display sucking and feeding difficulties [7]. The connection between infant suck and central nervous system integrity is highlighted by how it is controlled in the brainstem. NNS patterning is controlled by the suck central pattern generator (sCPG) housed in the brainstem reticular formation and is highly adaptable to mechanosensory inputs from the periphery. As such, suck can be altered by sensory experiences and deprivations, and is often used in therapeutic settings to enhance clinical outcomes such as growth, weight gain, state control and gastric motility [8–12]. Given the importance of NNS in early brainstem circuit formation and its relation to other important clinical outcomes, suck reflects an infant’s neuromotor function and requires an intact nervous system [13].

Prior research of preterm infants has linked neonatal NNS to later neurodevelopment in various domains, such as: motor skills, balance, intelligence, and language [14, 15]. In their 2015 study, Wolthuis-Stigter et al. (2015) used the Neonatal Oral-Motor Assessment Scale (NOMAS) to assess preterm infants’ suck patterns at 37 to 50 weeks post menstrual age (PMA) and the Bayley Scales of Infant and Toddler Development (Dutch version) at 2 years of age. They found that inability to sustain sucking and the absence of mature sucking patterns in young infants significantly increased the odds of abnormal neurodevelopmental outcomes at 2 years of age [15]. Further in 2017, Wolthuis-Stigter and colleagues examined a subset of the infants included in the previous study at 5 years of age. They tested if their sucking behaviors, sampled at 37 to 50 weeks’ PMA, were associated with their developmental outcomes at 5 years of age. Developmental outcomes were determined using various subtests from standardized assessments (e.g. Wechsler Preschool and Primary Scale of Intelligence, the Reynell Developmental Language Scales, etc.). Results showed that, the percentage of abnormal sucking patterns per child between 42 weeks’ and 50 weeks’ PMA were associated with language comprehension and intelligence at 5 years, and the age when a normal sucking pattern was achieved was associated with higher intelligence scores and improved motor outcome [14]. However, interpretation of results in both studies is limited due to a small sample size and a large portion of the sample having comorbidities (bronchopulmonary dysplasia and small for gestational age). Additionally, while the NOMAS is a useful tool to screen early oral-motor skills in infants, it relies on visual observation of NNS which is subjective, and studies of reliability and validity have found variable results [16, 17].

While these prior studies show connections between neonatal sucking and subsequent neurodevelopment, more research is needed to explore this relationship using a more quantitative and physiologically based approach to assess suck in full-term infants. Therefore, we aimed to determine if NNS dynamics at 3 months of age in full-term infants, sampled using our lab’s custom research pacifier, are associated with developmental outcomes, as measured by the Developmental Profile 3 (DP-3), at 12 months of age. We hypothesized that infants with NNS characterized by shorter burst duration, fewer cycles/burst and cycles/minute, higher amplitude, and more bursts at 3 months would have higher (better) scores on the DP-3 at 12 months. This hypothesis is based on prior research showing that as infants mature, they exhibit a more succinct NNS pattern with the characteristics detailed above [18].

## Methods

### Design

This study utilized a prospective design, with self-report and objective measures and was approved by Northeastern University’s Institutional Review Board (17-08-19). Caregivers provided written informed consent at the start of each study session on behalf of their infant. They were compensated with an Amazon gift card in exchange for their participation at each timepoint.

### Sample

These data were taken from a larger study (N = 85) examining the relation between sucking, feeding, and vocal development in the first year of life. This specific study examined a cohort of full-term infants at 3 and 12 months of age with no congenital or chromosomal anomalies. Participants were recruited between October 2017 and November 2020 by postings in caregiver Facebook groups, through posting flyers in the Greater Boston Area and through verbal communication. Sixty-seven infants (54% male) were evaluated at 3 months (average age 2.99 (0.27) months) and 12 months (average age 11.91 (0.26) months). All participants were deidentified. Study personnel had access to identifying information that was stored separately and in compliance with IRB policy.

### Study procedure

Study sessions were completed in the infant’s home environment at 3 and 12 months of age. Sessions began in the participant’s home approximately one hour before the infant’s anticipated feeding time. A five-minute NNS sample was collected at the 3-month session from the infant sucking on our lab’s custom research pacifier, described below. At the 12-month session, the DP-3 was administered through caregiver interview.

### Measurements

#### Non-nutritive suck

Our lab’s custom NNS device contains a Soothie pacifier (Philips Avent) attached to a handle, connected to a pressure transducer that transmits information to a data acquisition system (Power Lab, ADInstruments, Dunedin, New Zealand). The data acquisition system then connects to a laptop with LabChart software [19]. Calibration was completed before the study session. After calibration was completed, ensuring accuracy, caregivers were shown how to offer the infant the custom research pacifier. They were instructed to place the infant in a cradle hold in one hand and offer the pacifier with the other. Data were analyzed using LabChart software by trained study personnel who manually selected NNS bursts using criteria that have previously been used to examine NNS in young infants [20–22]. Selected bursts were then entered into a custom NNS Burst Macro, which allowed for quick processing of the following burst variables: duration (duration of the suck burst in seconds), frequency (intra-burst frequency measured in Hz), amplitude (measured as peak-height minus peak-trough in cmH_2_0), cycles/minute (number of peaks or cycles that occur in a minute), cycles/burst (number of peaks or cycles within a burst), bursts (number of bursts that occur in a minute). Next, the best two minutes of NNS data were selected based on cycle number, and minute rate averages for the outcome variables were attained from the two-minute sample.

#### Developmental profile 3

At the 12-month visit, study personnel administered the Developmental Profile 3 (DP-3) by interviewing the caregiver, in the home setting, who provided a yes/no response for each item [23]. The DP-3 assesses five key areas of development (Physical, Adaptive Behavior, Social-Emotional, Cognitive, and Communication) for children from infancy through age 12, and has been previously used in research to measure developmental outcomes [24, 25]. Each scale contains 34 to 38 items. The DP-3 takes approximately 20 minutes to administer (See Table 1 for domain descriptions). Each domain is summed to create a raw score, which is then converted into a standard score. Standard scores for the five domains are added together and converted into a General Development (total) score using the table in the DP-3 manual. Administration of each domain is discontinued after the caregiver reaches a ceiling of 5 “no” responses in a row. Cronbach’s alpha coefficient for the DP-3 was above 0.74 in all domains.

**Table 1:** DP-3 domain descriptions.

Developmental Profile-3: 180 items across 5 domains	
Domain Name	Domain Description
*Physical*	The Physical scale measures the development of fine and gross-motor skills. In infants, the skills assessed include the muscle coordination involved in head and core support, locomotion (rolling, crawling), and holding an object in their hands.
*Adaptive Behavior*	The Adaptive Behavior scale refers to skills that enable the child to cope with their environment, including self-care and survival behaviors. For infants, skills related to feeding and dressing are assessed.
*Social-Emotional*	The Social-Emotional scale assesses social and emotional competence in how the child engages with social situations. The child’s emotional understanding and ability to express their feelings and needs are examined when interacting with peers, family members, and other adults.
*Cognitive*	The Cognitive scale assesses the development of skills required for intellectual functioning in everyday life. In infants, skills assessed include memory, perception, and reasoning. Cognitive functioning is directly tied to abilities in other areas. As such, a child with a low Cognitive score may also show delays in other areas.
*Communication*	The Communication scale assesses the child’s understanding of spoken, written, and gestural language, as well as their ability to use verbal and nonverbal language to express themselves. Items in this scale are categorized under expressive and receptive communication.
*General Development Score (Total)*	The General Development Score is a composite of the five domains. It can be useful to provide a general sense of a child’s development in relation to their same-aged peers, however results should be interpreted in conjunction with individual domain scores in order to identify a child’s individual strengths and weaknesses.

Statistical analyses were performed using SPSS version 27.0. We used bivariate correlations to examine the association between NNS metrics and DP-3 domains and General Development score.

## Results

**Fig 2:**
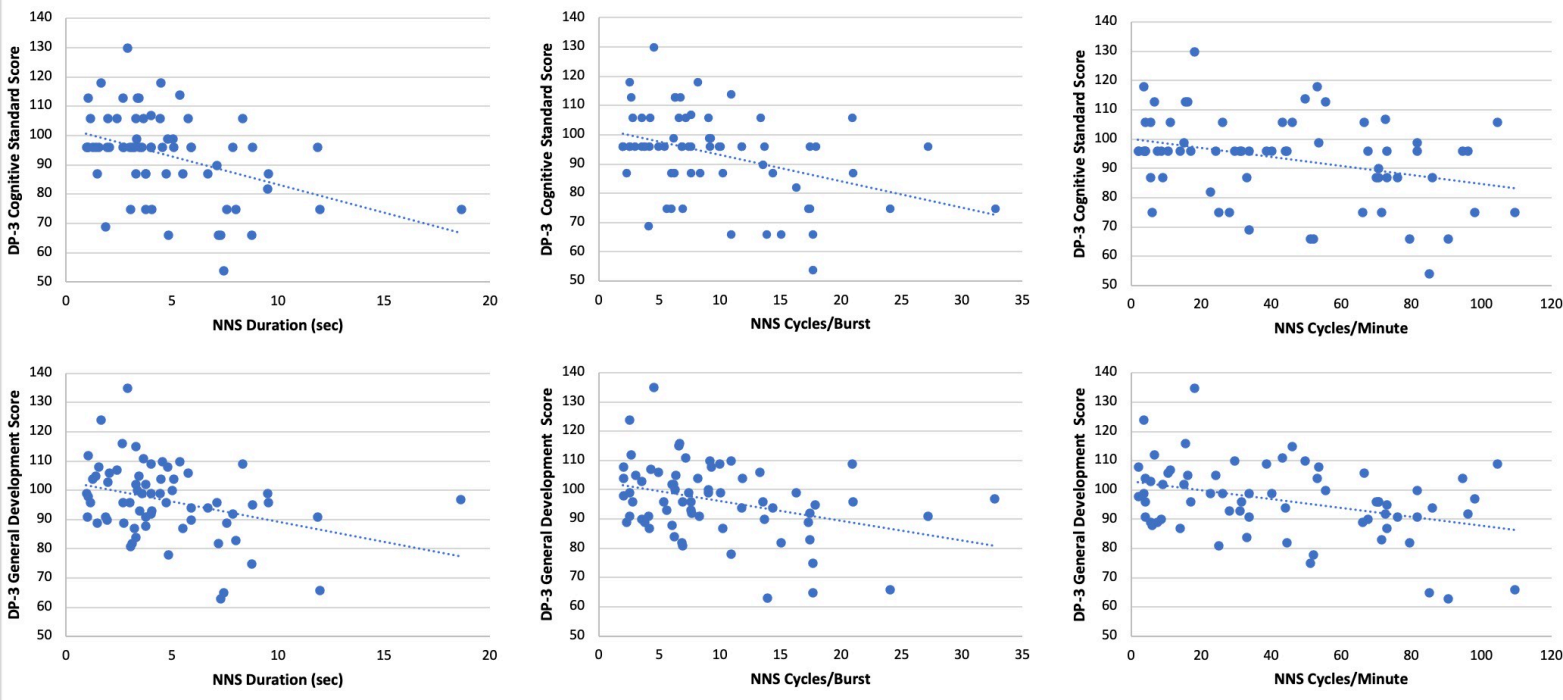
Significant correlations for NNS and DP-3. The first row shows the significant correlations between DP-3 Cognitive Standard score and NNS. The second row shows the significant correlations between DP-3 General Development score and NNS.

Sixty-seven full-term infants at 3- and 12-months of age were included (54% male), see Table 2 for infant and caregiver demographics. Infants were, on average, 2.99 (0.27) months of age at the 3-month time point and, on average, 11.91 (0.26) at the 12 month time point. A descriptive comparison of sex differences in DP-3 outcomes did not show any significant differences in the means and standard deviations between sexes. NNS duration was significantly negatively associated with DP-3 Cognitive score (r = -0.387, *p* = 0.001) and General Development score (r = -0.339, *p* = 0.005). NNS cycles/burst was significantly negatively associated with DP-3 Cognitive score (r = -0.369, *p* = 0.002) and General Development score (r = -0.348, *p* = 0.004). Lastly, NNS cycles/minute was significantly negatively associated with DP-3 Cognitive score (r = -0.298, *p* = 0.014) and General Development score (r = -0.371, *p* = 0.002). No other associations reached significance, see Table 3 and Fig 2.

**Table 2:** Infant and caregiver demographics.

***Infant Statistics (N = 67)***		
	3 Month Timepoint	12 Month Timepoint
Age in Months (SD)	2.99 (0.27)	11.91 (0.26)
Male	36 (54%)	
Birthweight in pounds (SD)	7.56 (1.00)	
Gestational Age (SD)	39.29 (1.14)	
Prior Pacifier Use (Yes)	49 (73%)	
***Caregiver Statistics (N = 134)***		
Married		130 (97%)
Education	College degree or higher	121 (91%)
	Master’s degree or higher	91 (68%)
Race	White	106 (81%)
	African American	4 (3%)
	Asian	18 (14%)
	Hispanic/Latino	2 (2%)
	Prefer not to Label	1 (1%)

*Note*: Education and race statistics were performed for all caregivers (N = 134). N = 1 caregiver was excluded from education statistics due to having missing data. N = 3 caregivers were excluded from the race statistics due to having missing data.

**Table 3:** All bivariate correlations for NNS and DP-3.

	NNS					
DP-3 Domains	Duration (sec)	Frequency (Hz)	Amplitude (cmH_2_0)	Burst Amount	Cycles/ Burst	Cycle/ Minute
Physical	-0.177	-0.019	0.090	-0.174	-0.212	-0.228
Adaptive Behavior	-0.163	-0.014	0.142	-0.068	-0.166	-0.189
Social-Emotional	-0.073	-0.074	-0.057	-0.188	-0.113	-0.209
Cognitive	**-0.413** **	-0.030	0.120	-0.077	**-0.401** **	**-0.331** **
Communication	-0.100	-0.056	0.027	-0.083	-0.087	-0.155
General Development (Total)	**-0.339** **	-0.018	0.111	-0.156	**-0.348** **	**-0.371** **

** Correlation is significant at the *p* < 0.01 level

In addition, correlations within DP-3 and NNS measures can be seen in S1 Fig and all data underlying the findings are available in S1 Dataset. The correlations within the NNS variables were consistent with previously reported associations [26, 27]. For instance, NNS cycles/burst and duration are highly correlated, but are both included in the analysis as some clinical sites may only count the number of cycles within a burst and others may count the duration of the burst. It is documented in the literature that domains within neurodevelopment can be interconnected [28], which is what was seen with the DP-3 measures.

## Discussion

This study aimed to determine if NNS sampled at 3 months of age was associated with developmental outcomes at 12 months of age. This is the first time that these associations have been examined using a quantitative and physiologic-based measure of NNS. Findings indicated that NNS burst duration, cycles/burst, and cycles/minute at 3 months of age were significantly negatively associated with the DP-3 Cognitive score and General Development score at 12 months. These findings imply that infants with more cycles/burst and per minute with a longer burst duration at 3-months had lower, or worse, scores on Cognitive and General Developmental outcomes at 12 months.

These findings were consistent with our hypothesis that infants who have more mature NNS at 3 months would have higher (better) scores on the DP-3 at 12 months. Our hypothesis was based on research from our lab that characterized NNS at 3 and 12 months of age and the change in NNS between these two timepoints [18]. Results showed that as infants mature across the first year of life, their NNS is characterized by fewer cycles/burst, cycles/minute and number of bursts, a shorter duration, and higher amplitude at 12 months. In this study, infants who exhibited these characteristics of more mature NNS patterning at 3 months of age had higher Cognitive and General Developmental outcomes at 12 months of age. Furthermore, these associations are congruent with prior research indicating that NNS is associated with subsequent neurodevelopment [14, 15]. Similar findings are also seen in the nutritive suck literature where Mizuno and Udea (2005) found that infants who exhibited poor nutritive suck coordination early in life were significantly more likely to demonstrate cognitive and complex motor delays at 18 months of age [29].

One potential reason we observed significant results in both Cognitive and General Development scores may be that many items on the Cognitive scale require skills in the other domains measured by the DP-3 (communication, motor, social and adaptive behaviors) [23]. Thus, this may demonstrate why an infant with lower scores in the Cognitive domain may exhibit lower General Development scores. No significant associations were observed between infant NNS variables and the Physical, Adaptive Behavior, Social-Emotional, and Communication domains. This may be due to the age that developmental outcomes were assessed because of the fewer items elicited at this timepoint compared to older ages. This study design allowed for infants to be followed to the 12-month timepoint; however, beyond 12 months we would likely see more significant changes in physical/motor complexity, expect infants to become more independent with adaptive behaviors (e.g. feeding and dressing), begin to express their socioemotional needs more clearly and communicate using words and signs. Interestingly, although not significant, a majority of the trends seen (with the exception of NNS amplitude) were in the negative direction, indicating that increased burst cycles that occur for a longer duration related to lower scores on the DP-3. More research is needed to examine these early trends further beyond the first year of life and into early childhood.

Findings from this work support emerging research highlighting the importance of assessing NNS in young infants. This study provides evidence that clinicians and developmental specialists (e.g. speech language pathologists, occupational therapists, physicians, etc.) can gain insight into an infant’s development through assessing their suck. This may highlight opportunities for earlier assessment of infants and more targeted therapies to improve developmental outcomes.

### Limitations

Potential limitations of this study need to be acknowledged. In this study, we used the DP-3 as our measure of development at the 12-month timepoint. The DP-3 is an accessible tool for clinicians and researchers to screen for potential delays in five key areas of development in as little as 20 minutes. However, it is important to note that it remains a screener, and further research should examine NNS in conjunction with more robust forms of neurological testing. Nevertheless, the succinctness and reliability of the DP-3 make these findings relatively easy to reproduce.

As we continue to expand upon this research, our next steps include adding additional study timepoints throughout the first year of life and beyond into early childhood. The addition of more timepoints will allow for testing of these preliminary results in more depth. Further, our sample was relatively homogenous and small (see Table 2) and therefore we did not include other covariates in our model, such as gestational age, birthweight, pacifier use, and caregiver education. As we add additional timepoints with a larger sample size, we will include covariates in statistical models. Additionally with a homogenous sample it is difficult to generalize these findings to special populations of infants. Research is currently underway to further characterize NNS in infants with conditions affecting feeding, such as premature infants and those with cleft lip and/or palate.

## Conclusions

Infants who had more NNS cycles per burst, more NNS cycles per minute, and longer burst duration at 3 months had lower Cognitive and General Development DP-3 scores at 12 months. This study provides evidence that developmental specialists should assess sucking behavior in young infants as an early marker of neurodevelopment.

## Supporting information

S1 Checklist STROBE statement—checklist of items that should be included in reports of observational studies. (DOCX)

S1 Fig Heatmap of correlations within DP-3 and NNS measures. Blue shade indicates higher correlations among variables and red shade indicated weaker correlations.
(TIF)

S1 Dataset Data file. This data file contains the NNS and DP-3 values underlying the findings described in the manuscript. Demographic data are kept confidential because the study uses personal data from minors.
(XLSX)
