# Supplementary material for: An examination of the association between infant non-nutritive suck and developmental outcomes at 12 months
Source: PLoS One. 2024 Feb 5;19(2):e0298016. doi: 10.1371/journal.pone.0298016 (PMC10843074; doi:10.1371/journal.pone.0298016)
Supplement: S1 Checklist — (DOCX) [file pone.0298016.s001.docx]

STROBE Statement—checklist of items that should be included in reports of observational studies

|  | Item No. | Recommendation | Page  No. | Relevant text from manuscript |
| --- | --- | --- | --- | --- |
| **Title and abstract** | 1 | (*a*) Indicate the study’s design with a commonly used term in the title or the abstract | 2 | This was a prospective study that utilized objective and self-report measures |
|  |  | (*b*) Provide in the abstract an informative and balanced summary of what was done and what was found | 2 | Researchers collected a five-minute NNS sample from 67 infants (54% male) at 3 months of age (average age 2.99 (0.27) months). At 12 months (average age 11.91 (0.26) months), researchers administered the Development Profile-3 through caregiver interview.  Infant NNS duration, cycles per burst, and cycles per minute were significantly negatively associated with the Development Profile-3 cognitive subsection and general scores at 12 months. |
| Introduction | | | |  |
| Background/rationale | 2 | Explain the scientific background and rationale for the investigation being reported | 6 | While these prior studies show connections between neonatal sucking and subsequent neurodevelopment, more research is needed to explore this relationship using a more quantitative and physiologically based approach to assess suck in full-term infants. |
| Objectives | 3 | State specific objectives, including any prespecified hypotheses | 6 | Therefore, we aimed to determine if NNS dynamics at 3 months of age in full-term infants, sampled using our lab’s custom research pacifier, are associated with developmental outcomes, as measured by the Developmental Profile 3 (DP-3), at 12 months of age. We hypothesized that infants with NNS characterized by shorter duration, fewer cycles/burst and cycles/minute, higher amplitude, and more bursts at 3 months would have higher (better) scores on the DP-3 at 12 months. |
| Methods | | | |  |
| Study design | 4 | Present key elements of study design early in the paper | 6 | This study utilized a prospective design, using self-report and objective measures and was approved by Northeastern University’s Institutional Review Board |
| Setting | 5 | Describe the setting, locations, and relevant dates, including periods of recruitment, exposure, follow-up, and data collection | 7 | Researchers performed study sessions in the infant’s home environment at 3 and 12 months of age.  Participants were recruited between 2017 and 2020 by postings in caregiver Facebook groups, through posting flyers in the Greater Boston Area and through word of mouth. |
| Participants | 6 | (*a*) *Cohort study*—Give the eligibility criteria, and the sources and methods of selection of participants. Describe methods of follow-up  *Case-control study*—Give the eligibility criteria, and the sources and methods of case ascertainment and control selection. Give the rationale for the choice of cases and controls  *Cross-sectional study*—Give the eligibility criteria, and the sources and methods of selection of participants | 7 | This specific study examined a cohort of full-term infants at 3 and 12 months of age with no congenital or chromosomal anomalies. |
|  |  | (*b*) *Cohort study*—For matched studies, give matching criteria and number of exposed and unexposed  *Case-control study*—For matched studies, give matching criteria and the number of controls per case |  | N/A |
| Variables | 7 | Clearly define all outcomes, exposures, predictors, potential confounders, and effect modifiers. Give diagnostic criteria, if applicable | 7, 8 | A five-minute NNS sample was collected at the 3 month session from the infant sucking on our lab’s custom research pacifier. At the 12 month session, researchers administered the DP-3 through caregiver interview  NNS variables: duration (duration of the suck burst in seconds), frequency (intra-burst frequency measured in Hz), amplitude (measured as peak-height minus peak-trough in cmH_2_0), cycles/minute (number of peaks or cycles that occur in a minute), cycles/burst (number of peaks or cycles within a burst), bursts (number of bursts that occur in a minute).  The DP-3 assesses five key areas of development (Physical, Adaptive Behavior, Social-Emotional, Cognitive, and Communication). Standard scores for the five subsections are combined and converted into a General Development (total) score |
| Data sources/ measurement | 8* | For each variable of interest, give sources of data and details of methods of assessment (measurement). Describe comparability of assessment methods if there is more than one group | 7, 8, 9 | Measurements section – NNS and DP-3 |
| Bias | 9 | Describe any efforts to address potential sources of bias | 7 | All participants were deidentified |
| Study size | 10 | Explain how the study size was arrived at | 7 | Sixty-seven infants (54% male) were evaluated at 3 months (average age 2.99 (0.27) months) and 12 months (average age 11.91 (0.26) months). |

Continued on next page

| Quantitative variables | 11 | Explain how quantitative variables were handled in the analyses. If applicable, describe which groupings were chosen and why | 10 | We used bivariate correlations to examine the association between NNS metrics and DP-3 subsections and General Development score. |
| --- | --- | --- | --- | --- |
| Statistical methods | 12 | (*a*) Describe all statistical methods, including those used to control for confounding | 10 | We used bivariate correlations to examine the association between NNS metrics and DP-3 subsections and General Development score. |
|  |  | (*b*) Describe any methods used to examine subgroups and interactions |  | N/A |
|  |  | (*c*) Explain how missing data were addressed |  | N/A – No missing data expect for N=4 caregiver demographics |
|  |  | (*d*) *Cohort study*—If applicable, explain how loss to follow-up was addressed  *Case-control study*—If applicable, explain how matching of cases and controls was addressed  *Cross-sectional study*—If applicable, describe analytical methods taking account of sampling strategy |  | N/A – all participants participated in both timepoints |
|  |  | (*e*) Describe any sensitivity analyses |  | N/A |
| Results | | | | |
| Participants | 13* | (a) Report numbers of individuals at each stage of study—eg numbers potentially eligible, examined for eligibility, confirmed eligible, included in the study, completing follow-up, and analysed | 10 | Sixty-seven full-term infants at 3- and 12-months of age were included (54% male), see Table 2 for infant and caregiver demographics. |
|  |  | (b) Give reasons for non-participation at each stage |  | N/A |
|  |  | (c) Consider use of a flow diagram |  | N/A |
| Descriptive data | 14* | (a) Give characteristics of study participants (eg demographic, clinical, social) and information on exposures and potential confounders | 11 | Table 2 . Infant and Caregiver Demographics |
|  |  | (b) Indicate number of participants with missing data for each variable of interest |  | N/A |
|  |  | (c) *Cohort study*—Summarise follow-up time (eg, average and total amount) |  | N/A – all participants completed both study sessions |
| Outcome data | 15* | *Cohort study*—Report numbers of outcome events or summary measures over time | 11, 12 | See Table 2 and 3 for summary |
|  |  | *Case-control study—*Report numbers in each exposure category, or summary measures of exposure |  |  |
|  |  | *Cross-sectional study—*Report numbers of outcome events or summary measures |  |  |
| Main results | 16 | (*a*) Give unadjusted estimates and, if applicable, confounder-adjusted estimates and their precision (eg, 95% confidence interval). Make clear which confounders were adjusted for and why they were included | 12 | Findings indicated that NNS duration, cycles/burst, and cycles/minute at 3 months of age were significantly negatively associated with the DP-3 Cognitive score and General Development score at 12 months. These findings imply that infants with more cycles/burst and per minute with a longer burst duration at 3-months had lower, or worse, scores on Cognitive and General Developmental outcomes at 12 months. These findings were consistent with our hypothesis that infants who have shorter, more succinct NNS at 3 months would have higher (better) scores on the DP-3 at 12 months. |
|  |  | (*b*) Report category boundaries when continuous variables were categorized |  |  |
|  |  | (*c*) If relevant, consider translating estimates of relative risk into absolute risk for a meaningful time period |  |  |

Continued on next page

| Other analyses | 17 | Report other analyses done—eg analyses of subgroups and interactions, and sensitivity analyses |  | N/A |
| --- | --- | --- | --- | --- |
| Discussion | | | | |
| Key results | 18 | Summarise key results with reference to study objectives | 13 | In this study, infants who exhibited these characteristics of more mature NNS patterning at 3 months of age had higher Cognitive and General Developmental outcomes at 12 months of age. Furthermore, these associations are congruent with prior research indicating that NNS is associated with subsequent neurodevelopment |
| Limitations | 19 | Discuss limitations of the study, taking into account sources of potential bias or imprecision. Discuss both direction and magnitude of any potential bias | 14 | Potential limitations of this study need to be acknowledged – limitations section |
| Interpretation | 20 | Give a cautious overall interpretation of results considering objectives, limitations, multiplicity of analyses, results from similar studies, and other relevant evidence | 14 | Findings from this work support emerging research highlighting the importance of assessing NNS in young infants. This study provides evidence that clinicians and developmental specialists (e.g. speech language pathologists, occupational therapists, physicians, etc.) can gain insight into an infant’s development through assessing their suck. This may highlight opportunities for earlier assessment of infants and more targeted therapies to improve developmental outcomes. |
| Generalisability | 21 | Discuss the generalisability (external validity) of the study results | 14 | Nevertheless, the succinctness and reliability of the DP-3 make these findings relatively easy to reproduce. |
| Other information | |  | | |
| Funding | 22 | Give the source of funding and the role of the funders for the present study and, if applicable, for the original study on which the present article is based |  | Grant #: DC016030, The National Institute on Deafness and Other Communication Disorders The funders had no role in the study  design, data collection and analysis, decision to publish or preparation of the manuscript. |

*Give information separately for cases and controls in case-control studies and, if applicable, for exposed and unexposed groups in cohort and cross-sectional studies.

**Note:** An Explanation and Elaboration article discusses each checklist item and gives methodological background and published examples of transparent reporting. The STROBE checklist is best used in conjunction with this article (freely available on the Web sites of PLoS Medicine at http://www.plosmedicine.org/, Annals of Internal Medicine at http://www.annals.org/, and Epidemiology at http://www.epidem.com/). Information on the STROBE Initiative is available at www.strobe-statement.org.
