# Supplementary figures and images for: An examination of the association between infant non-nutritive suck and developmental outcomes at 12 months
Source: PLoS One. 2024 Feb 5;19(2):e0298016. doi: 10.1371/journal.pone.0298016 (PMC10843074; doi:10.1371/journal.pone.0298016)

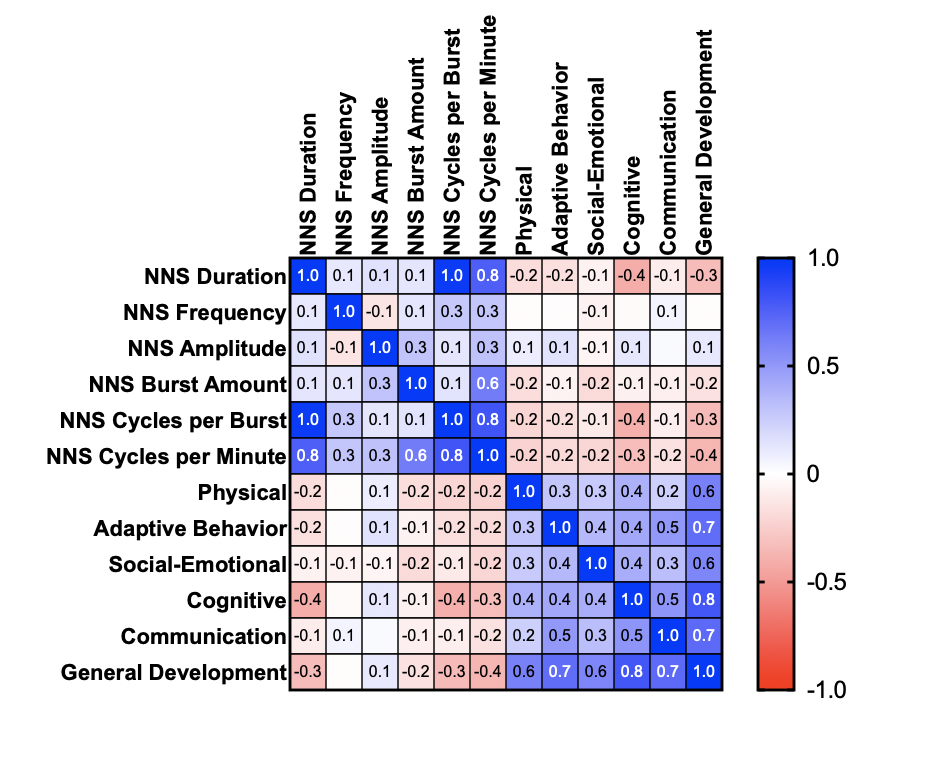

Supplement: S1 Fig — Blue shade indicates higher correlations among variables and red shade indicated weaker correlations. (TIF) [file pone.0298016.s002.tif]
